# Renaming NAFLD to MAFLD: Could the LDE System Assist in This Transition?

**DOI:** 10.3390/jcm10030492

**Published:** 2021-01-31

**Authors:** Amedeo Lonardo

**Affiliations:** Department of Internal Medicine, Azienda Ospedaliero-Universitaria di Modena, 4110 Modena, Italy; lonardo.amedeo@aou.mo.it

**Keywords:** classification, endocrine, hepatocellular carcinoma (HCC), LDE system, NAFLD, MAFLD, metabolic syndrome, psycho-depression, virus-associated fatty liver disease (VAFLD)

## Abstract

Our understanding of fatty liver syndromes and their relationship with the metabolic syndrome has improved over recent decades and, paralleling this, we are now at the dawn of the NAFLD (nonalcoholic fatty liver disease) to MAFLD (metabolic-associated fatty liver disease) transition. The pitfalls of NAFLD diagnosis, together with disappointing results in therapeutic trials, and the inconsistencies and risks inherent in a “negative” definition (such as “nonalcoholic”) as opposed to a “positive” one (i.e., “metabolic”) are predicted to facilitate the proposed renaming of NAFLD to MAFLD. However, a premature change of terminology would not necessarily address major unmet needs in this area, and may even become counterproductive. As an aid to selecting more homogeneous cohorts of patients, I propose the LDE (Liver, Determinants, Extra-hepatic) classification system which, in principle, may help to assess the natural course of disease as well as the efficacy of novel drugs in patients with NAFLD/MAFLD.

## 1. Background, Aim and Method

Steatosis, i.e., the pathological accumulation of intra-hepatic fat content, has been known since 1845 thanks to the work by Addison, who described liver histology changes induced by alcohol [1]. In 1938, Connor pinpointed the potential for fatty liver disease, owing to either alcohol or diabetes, to progress to liver cirrhosis [2] and, in 1964, Dianzani clearly addressed the pathogenesis of steatosis [3]. However, it was not until the 1980s that the terms “nonalcoholic steatohepatitis” (NASH) and “nonalcoholic fatty liver disease” (NAFLD) were coined by Ludwig et al. [4], and Shaffner and Thaler [5], respectively. Following decades of research, we are now fully aware that NAFLD and NASH are pathogenically diverse, are common in the general population on a worldwide basis, exact a heavy toll in terms of medical-related as well as indirect expenditures and remain orphans of an effective and safe drug treatment [6,7,8,9]. Of concern, many NASH trials fail [10], suggesting that we are far from dominating this non-transmissible though epidemic liver disease. Recently, based on previous suggestions reviewed in [11], it has been proposed that NAFLD should be renamed as MAFLD, i.e., metabolic-(associated) fatty liver disease [12,13]. With this background, the present review aims at illustrating the reasons underlying the debate on nomenclature of common fatty liver syndromes while highlighting unanswered research questions. Finally, I will illustrate a novel proposal of classification, the so called LDE (Liver, Determinants, Extra-hepatic) system, which may integrate both NAFLD and MAFLD for better pheno-genotyping of individual patients and cohorts. With the aim of analyzing published studies, the Pub Med data base was consulted using the following key-words: NAFLD, NASH, and MAFLD, without date limitations. Only references that were deemed to be relevant to the topic were retained.

## 2. NAFLD, Diagnosis, Pitfalls and Classification

NAFLD, comprising the whole gamut of alcohol-like liver lesions (i.e., steatosis, steatohepatitis with/without fibrosis, cirrhosis and hepatocellular carcinoma (HCC)) though observed in the nonalcoholic [14], was formerly believed to be “the hepatic manifestation of the Metabolic Syndrome”. However, this old notion is, at best, incomplete and accumulating data strongly indicate that the association of NAFLD with Metabolic Syndrome is indeed mutual and bi-directional [15,16,17].

The diagnosis of NAFLD is based on the non-invasive identification of fatty changes in the liver (through biomarkers and/or imaging studies) in the absence of competing causes of (steatogenic) liver disease [18]. Compared to biomarkers and other imaging techniques (such as Computed Tomography scanning and Magnetic Resonance) conventional ultrasonography retains a key role in as much as it is cheap, repeatable, widely available, allows the ruling out of focal liver disease and permits those semi-quantitative evaluations that mirror metabolic derangements and liver histology changes [19,20]. Liver biopsy is the diagnostic standard, given that it provides a definite characterization of the elementary histological lesions: steatosis, ballooning, inflammation and fibrosis, thereby permitting the differentiation of the more indolent uncomplicated steatosis from the more rapidly progressive NASH forms [21,22]. Pros and cons of the two chief histological methods of assessment including the American NASH Clinical Research Network (CRN) and the European Fatty Liver Inhibition of Progression (FLIP) have been discussed analytically elsewhere [23]. Importantly, neither the CRN nor the FLIP system evaluate portal chronic inflammation and the presence and location of Mallory-Denk bodies, which may raise the point of the differentiation of NAFLD from viral hepatitis, cholestatic disease and Wilson’s disease [24]. Moreover, the use of a numerical histological endpoint in the NASH clinical trials (typically the decrease of NAFLD activity score (NAS) by ≥2, without any worsening of fibrosis stage) will not necessarily guarantee the resolution of NASH [24]. The histological distinction of cases of NAFLD from those owing to competing liver diseases, such as drug-induced liver injury, congestive hepatopathy and lysosamial acid lipase deficiency, represent additional pitfalls [25]. Concurrent autoimmune hepatitis and NASH may be quite hard to distinguish histologically and steroid administration may improve the histological activity of autoimmune hepatitis while worsening NASH [26].

Per classification (Table 1), NAFLD is defined as “primary” when it is associated with (or deemed to herald an incidence of) Metabolic Syndrome [27,28,29]. “Secondary” NAFLD forms are myriad and include, among others, diseases occurring as a result of nutritional disorders, complications of abdominal surgery, use of several drugs, occupational exposure to organic solvents and (rare) metabolic disorders [27,30]. In addition, NAFLD may often occur secondarily to common viral infections (the so called “virus-associated fatty liver disease” or VAFLD) [31] and frequently occurring endocrine derangements [32]. These secondary NAFLD forms need to remain separated from primary NAFLD given that, for example, VAFLD owing to HIV infection follows a worse course than primary NAFLD [33] and that NAFLD associated with hypothyroidism has a specific pathogenesis which, in theory, is amenable to complete reversal following thyroid hormone replacement therapy [34].

NAFLD exacts a heavy toll on global medical expenditures. Younossi reported that the direct medical costs related to NAFLD per year were, in the United States, about $103 billion and, in four European countries alone (Germany, France, Italy, and United Kingdom), it totaled about €35 billion [35].

## 3. The Metabolic Syndrome

The development of Metabolic Syndrome, which defines the cluster of multiple cardio-metabolic derangements including visceral obesity, dyslipidemia, arterial hypertension, and type 2 diabetes mellitus, is deemed to be affected by those lifestyle habits which are typical of industrialized countries and also adopted by many developing countries [36]. Metabolic Syndrome is triggered by visceral obesity [37] which, in its turn, results from a positive energy balance owing to poor diets and a sedentary lifestyle [38], together with an as yet incompletely defined polygenic hereditary asset [39]. Insulin resistance is central in the pathogenesis of Metabolic Syndrome [40]. This is not to say that the ultimate mechanisms which are eventually conducive to the development of visceral adiposity and insulin resistance are limpidly clear and indeed it is possible that central dysregulation plays a key role [41]. Confirming this, a recent study found that that high brain insulin sensitivity anticipates weight loss during lifestyle intervention and is associated with a favorable body fat distribution; additionally, high brain insulin sensitivity is also associated with less regain of fat mass during a nine-year follow-up [42].

In many animals, including mammals, the regulation of reproductive, metabolic and behavioral activities follows a cyclical pattern of responses. Light exposure is believed to orchestrate such responses through the regulation of melatonin secretion [43]. For example, facilitated by an abundance of nutrients during longer summer days, hibernating mammals adopt those behavioral changes (such as decreased sleep length and maximized food intake) properly suited to storing energy that will rapidly result in a state of insulin resistance and altered secretion of adiponectin and leptin [43]. Conversely, during longer winter nights, increased melatonin secretion, which is regulated via the suprachiasmatic nucleus, will enhance insulin sensitivity and energy availability whilst the animal is dormant [43]. It is conceivable that human diseases possibly recapitulate animal behavior and, if this is the case, that the Metabolic Syndrome may mimic a “pre-hibernation” state reflecting dysregulation of neural pathways. This innovative pathogenic perspective paves the way for a line of research addressing deregulated serotonin pathways and psycho-depression among those with nonalcoholic fatty liver disease (NAFLD) [44,45,46]. For example, a subset of well characterized patients might be submitted to psychological counseling to disclose and address those offending life events and traumatic experiences that will eventually be conducive to depression in predisposed individuals. Other individuals with NAFLD might conceivably be administered antidepressants, though in the setting of randomized controlled trials (RCTs) on NASH, while keeping in mind that this class of drugs may often bear unfavorable metabolic effects.

Irrespective of its intimate patho-mechanisms, Metabolic Syndrome exacts a heavy toll on medical expenditures [47].

## 4. NAFLD and the Metabolic Syndrome: Chicken or Egg?

As alluded to above, a consistent body of evidence supports the notion that a bi-directional relationship links NAFLD with (features of) Metabolic Syndrome, with insulin resistance being the shared common pathophysiological denominator [48]. In the medical literature, the historical “chicken and egg” debate regarding the chronological association of NAFLD with Metabolic Syndrome, [41] eventually found its answer through novel data showing that NAFLD is both the cause and the effect of the Metabolic Syndrome (reviewed in [16,17]). However, it soon became clear that addressing the key pathogenic determinants of NASH would not necessarily improve disease outcomes. One of the first lines of evidence regarding this was provided by the finding that insulin sensitizers failed to invariably reverse NASH in all cases, did not reverse or even worsened mitochondrial abnormalities in NASH and, conversely, histological improvement, at least in some patients, was observed with pharmacological agents, such as vitamin E, acting through mechanisms other than insulin sensitization [49].

Why then, in overt conflict with studies on pathogenesis, is the correction of insulin resistance invariably not sufficient to successfully treat NASH in the majority of patients? This probably results from varying pathogenic mechanisms, concurring to determine liver damage to a variable extent in the individual patients. Based on this notion, treatment should be tailored to the individual subject [49]. However, how this can be accomplished is far from established.

A variety of pathogenic mechanisms interactively take part in the development of NAFLD/NASH and the identification of the role of each in the individual patient is an unmet research and clinical challenge. Just to provide a few examples, should individuals with NAFLD in the absence of Metabolic Syndrome [50] and those with lean NAFLD [51] be treated in a similar way to those with obesity? Should men and women be treated alike despite this not being evidence-based? [52].

**Figure 1:**
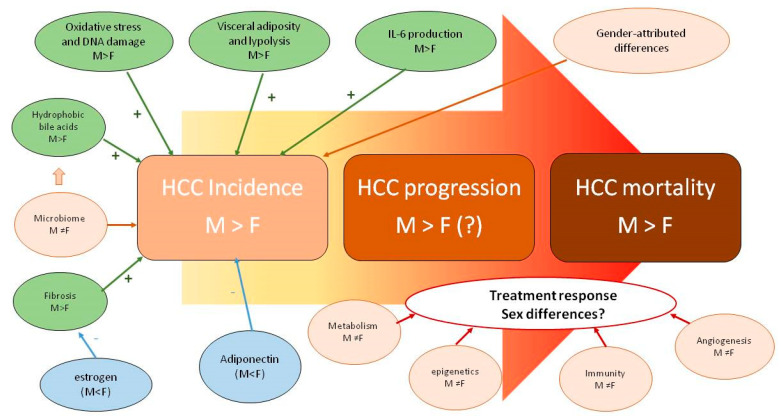
Sex disparity in pathobiology, epidemiology and clinical features of HCC (reprinted from [53]).

Irrespective of whether NAFLD is the cause or the effect of Metabolic Syndrome (and we now know it is both), it is worth highlighting that these two conditions are synergistic risk factors for the development of HCC [48]. HCC represents the most common primary liver cancer and is the fourth most common cause of mortality owing to cancer [53]. The reasons underlying differences in the male-to-female ratio of the incidence of HCC (which ranges between 2–3 in most countries) are intriguing and incompletely understood (Figure 1).

The biological grounds underlying sex disparity in the incidence, progression and mortality of HCC are incompletely understood and probably related to multiple factors pertaining to individual life habits and endocrine-metabolic factors, as well as to cancer biology per se.

Interestingly, similar to HCC, NAFLD and the Metabolic Syndrome have distinct features of sexual dimorphism which include the prevalence and significance of dysglycemia (impaired fasting glucose versus impaired glucose tolerance); sexual dimorphism in body fat distribution and patterns of android and gynoid obesity; pathobiology of adipocytes including cell size and function; and the influence of estrogen decline on clustering risk factors and the inherent dangers of liver fibrosis progression [52,54,55].

In their seminal article, voicing concerns from an established pipeline of previous studies, Bellentani and Tiribelli properly identified the limitations included in the “negative” definition of NAFLD and NASH as opposed to a positive one, i.e., “metabolic” [56]. In agreement, Fouad et al. also pinpointed that the allusion to alcohol contained in the term “nonalcoholic” incurred the risks of trivialization, stigmatization and non-consideration from health authorities [11]. Adding to the above limitations and risks, the impressive number of “therapeutic casualties” of innovative drugs in the NASH trials arena including simtuzumab, selnsertib, emricasan and MSDC-0602K, calls for careful consideration [57,58,59,60].

The reasons underlying this cemetery of failures are undoubtedly multiple and include the variability in endpoints adopted over time [61], as well as disease modifiers such as diet and exercise [62]. Ratziu and Friedman have recently argued that factors that may impact on the outcomes of NASH trials should be differentiated into NASH-cirrhosis as opposed to non-cirrhotic disease, and in pre-clinical vs. clinical studies; that some of these failures result from the overly simplistic interpretation of findings from small-sized pilot studies; and that the identification of primary versus secondary end-points may be confusing and the effects of alcohol and placebo uncertain [10]. It is also clear, in my opinion, that cohorts of NASH patients enrolled in RCTS are homogenous only with regard to liver biopsy findings rather than for determinants and extra-hepatic features of disease (such as those more extensively discussed below). This may be a strong limitation given that any given liver histology finding in the individual patient is the final result of often substantially differing spectra of pathogenic and systemic scenarios which may potentially affect, more than liver histology per se, response to treatments. In other words, liver histology compatible with NASH may underly a variable proportion of different contributions resulting from sex, genetics, endocrine modifiers, lifestyle habits, and concurrent extra-hepatic clinical phenotypes which, taken collectively, have the potential to alter the natural course of liver disease in the individual patient. All the considerations illustrated above, including the pitfalls in NASH diagnosis and the inconsistencies and the risks of a negative definition, together with the inability of the NAFLD terminology to provide homogeneous cohorts of NASH patients to submit to RCTs, strongly support the notion that a change in NAFLD terminology is an urgent and unmet research priority.

## 5. The NAFLD-MAFLD Debate

In trying to incorporate those proposals regarding the inaccuracy and possible negative consequences of using the term “NAFLD” that have accumulated over the past twenty years, a panel of experts from as many as 22 countries has recently proposed novel names and definitions for NAFLD in adults—namely, metabolic dysfunction-associated fatty liver disease (MAFLD) [12,13]. This proposal has rapidly gained consensus in Latin America, North Africa and the Middle East [62,63], indicating that the motivations to abandon the old nosography are universally believed to outnumber the reasons for maintaining it.

MAFLD is defined as the presence of hepatic steatosis (detected either histologically or by imaging techniques) in those individuals who have either type 2 diabetes or obesity. Interestingly, the presence of at least two, among the following criteria: abnormal abdominal adiposity (assessed with waist circumference above the sex-specific and ethnicity-specific threshold); arterial hypertension; hypertriglyceridemia; low HDL-cholesterol; pre-diabetes; insulin resistance (HOMA-IR); and subclinical systemic inflammatory state (high-sensitivity C-Reactive Protein), is deemed to be equivalent to either obesity or diabetes. It remains unproven whether NAFLD in the diabetic patient will follow the same course as in the metabolically healthy obese. Similarly, it remains to be seen whether individuals with borderline metabolic derangements will be prone to the same risk of developing those hepatic and extra-hepatic complications that we commonly find in association with overt diabesity. From the histological point of view, NAFLD and NASH were more rigorously defined [21] than MAFLD and defining liver histology remains a milestone in our capacity to predict clinical outcomes of disease [64,65]. However, clinicians and patients will undoubtedly appreciate the possibility of diagnosing MAFLD non-invasively given the many criticisms that can be attributed to liver biopsy [44]. Whether, and to what extent, steatosis/steatohepatitis/fibrosis seen in a dysmetabolic individual is MAFLD rather than “alcoholic-and-nonalcoholic liver disease” remains uncertain [66].

The panel of experts also issued a set of diagnostic criteria to establish the diagnosis of MAFLD- related cirrhosis, so avoiding the use of the term cryptogenic cirrhosis among dysmetabolic individuals [12,13]. Given that fatty changes may disappear over time [67], the panel suggested that patients with established cirrhosis, though in the absence of histological evidence of steatohepatitis, should be considered to have MAFLD-cirrhosis if they meet at least one of the following criteria: past or present evidence of dysmetabolic traits that satisfy the criteria to diagnose MAFLD (as reported above) with at least one of the following criteria in their medical history, namely previous biopsy-proven MAFLD, or previous evidence of hepatic steatosis via imaging techniques [12,13]. In this connection, it is worth remembering the seminal study in 1999 in which Caldwell, based on his personal series of 70 cases, was the first to suggest that “NASH plays an under-recognized role in many patients with cryptogenic cirrhosis, most of whom are older, type 2 diabetic and obese females” [68].

Although probably not the ultimate answer to all unmet clinical needs, the definition of MAFLD goes one step further in the attempt to better define NAFLD patients [66]. Indeed, the name “MAFLD” progresses from a “negative” (nonalcoholic) to a “positive” (metabolic-associated) qualification of fatty liver syndromes. Moreover, it is logical to differentiate NAFLD associated with (i.e., MAFLD) or dissociated from Metabolic Syndrome (i.e., genetic NAFLD), given that either may follow different outcomes, such as extensively discussed below. Moreover, the novel definition of MAFLD utilizes the lessons learnt regarding the ominous interaction of NAFLD with Metabolic Syndrome, an association which worsens liver histology, facilitates fibrosis progression, exposes to the risk of developing HCC and decreases life expectancy of patients with NAFLD [69,70,71,72]. However, the road ahead remains long given that, for example, we still know little, if anything, regarding the impact of other determinants of disease such as sex and gender [52], gut microbiota [73], the role of hyper-ferritinemia [74,75] and of genetic polymorphisms [76].

## 6. The LDE Classification System

**Figure 2:**
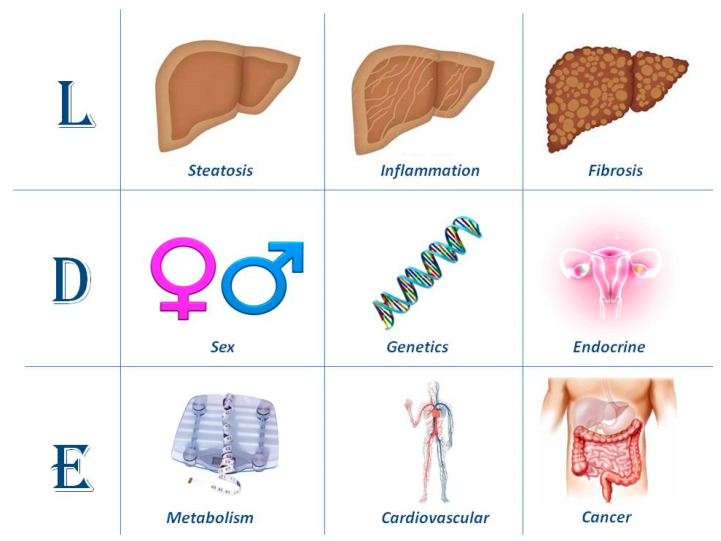
Articulating the taxonomy of nonalcoholic fatty liver disease (NAFLD)/metabolic-associated fatty liver disease (MAFLD) with the LDE system. This schematic figure illustrates the proposed high standard of accuracy in defining NAFLD/MAFLD cases to be recruited for trials of innovative treatment, as well as for a better prediction of the natural course of disease in the individual patient. In its essence, the system requires clarification of nine criteria that should be recorded to better characterize cohorts of patients recruited in randomized controlled trials.

On the background of the difficulties and complexities illustrated so far, it would appear logical to be as accurately descriptive as possible in defining the hepatic features of disease, its determinants and extra-hepatic involvement. The so called LDE system classification (Figure 2) [44] is an example of how this may be accomplished in the individual patient.

Lonardo and Ballestri [44] have recently proposed the so called LDE system (Liver, Determinants and Extra-hepatic). The first section of the LDE syntax is the prefix “L” for “Liver” which identifies the key histological determinants of disease. While this section may be relatively straightforward to address based on liver histology findings, it can also be characterized noninvasively. For example, the extent of steatosis may accurately be gauged through controlled attenuation parameter (CAP) or, whenever available, Magnetic Resonance-based techniques [77,78,79]; and the stage of fibrosis with elasto-graphic techniques (based on either ultrasonography or Magnetic Resonance) or with biomarkers [80,81]. Liver inflammation may also be noninvasively evaluated with various either traditional or innovative biomarkers, the most widely available and best known being liver enzymes [80,81,82,83], although transaminases have a low sensitivity for the diagnosis of advanced NAFLD forms [84].

The central section of the LDE system is “D”, which stands for the “Determinants” of the fatty liver syndrome in the individual patient. These include, for example, sex and menopausal status; the presence of specific single nucleotide polymorphisms (SNPs) known to be associated with certain disease outcomes; and endocrine conditions deemed to facilitate the development of secondary forms of fatty liver syndromes. An exhaustive analysis of each of these determinants is beyond the scope of the present review. Moreover, recent studies have already covered the importance of sex and the endocrine system in the development and progression of NAFLD [32,85]. Here we will discuss selected studies supporting the notion that the presence of certain SNPs may affect either the natural course of NAFLD or its response to treatment.

One of the first studies suggesting that metabolic NAFLD (i.e., what we would now call MAFLD) could be different from “genetic NAFLD” was conducted by Lonardo et al. in 2006. By evaluating a small cohort of 22 individuals with NAFLD owing to familial heterozygous hypobetalipoproteinemia compared to 48 who had metabolic NAFLD and 42 healthy NAFLD-free controls, these authors found that individuals with metabolic NAFLD had higher levels of insulin resistance (as assessed with HOMA-IR) and gamma-glutamyl transferase than those in whom NAFLD was associated with familial heterozygous hypobetalipoproteinemia; this last cohort exhibited a level of insulin resistance in the same order of magnitude as found among healthy controls [86]. Bringing this further, Di Costanzo et al. accurately characterized a cohort of 83 blood donors with the mutant GG genotype (group G), 100 patients with features of the Metabolic Syndrome but the wild type CC genotype (group M), and 74 blood donors with the wild type CC genotype (controls) in the patatin-like phospholipase domain-containing 3 gene (*PNPLA3*). These authors found that, following adjustment for confounding factors, the median carotid intima-media thickness (a widely used marker of subclinical atherosclerosis) in group M was significantly greater than that in group G (0.84 (0.70–0.95) mm vs. 0.66 (0.55–0.74) mm; *p* < 0.001), and the latter was not different from that of controls (0.70 (0.64–0.81) mm) suggesting that hepatic steatosis was associated with an increased risk of subclinical carotid atherosclerosis burden only in patients with Metabolic Syndrome rather than in genetic NAFLD owing to *PNPLA3* SNP [87]. Confirming this pioneer study, Käräjamäki et al. followed 249 patients with NAFLD (diagnosed based on liver ultrasonography) comprised in a large cohort of 958 middle-aged Finns for 21 years. Data have shown that Metabolic Syndrome, rather than the gene polymorphisms studied (*PNPLA3* rs738409, *TM6SF2* rs58542926 and *MBOAT7* rs641738), predicted an increased mortality owing to either overall causes or cardiovascular diseases among NAFLD subjects [88]. As regards the risks of fibrosis progression and of developing HCC, Singal et al. have clearly shown in their meta-analytic review that *PNPLA3* rs738409 confers an increased risk of advanced fibrosis in individuals in whom chronic liver disease occurs as a result of varying etiologies, notably including NAFLD. Moreover, these authors also found that *PNPLA3* was associated with an increased risk of developing HCC in those with NASH [89]. Similarly, also the *MBOAT7-TMC4* variant rs641738 has been found to increase the risk of HCC in NAFLD patients [90]. Pillai et al., by evaluating a large series of individuals with type 2 diabetes, of whom 1822 were treated with basal insulin peglispro and 1270 with insulin glargine, found that those with the *PNPLA3* (148M/M) genotype treated with basal insulin peglispro were more prone to developing an increased intrahepatic fat content assessed with Magnetic Resonance Imaging [91]. More recently, Chen et al. have elegantly shown that *PNPLA3* I148M might modify the anti-NAFLD response to exenatide based on in vitro and clinical evidence [92]. Collectively, all of the above studies support the importance of addressing the most common SNPs affecting the risk of development and progression of NAFLD [76,93].

Finally, the suffix “E” stands for “extra-hepatic” manifestations of fatty liver syndromes, the most common of which are metabolic, cardiovascular and cancer, extensively reviewed elsewhere by this group and others [94,95,96,97,98]. The extent and accuracy of the assessment which should be carried out in the individual patient to fully characterize the “E” section of the LDE system is best tailored based on careful evaluation of personal and family history.

The LDE classification system is intended to integrate and not replace the existing nomenclature in the attempt to better define the histological, pathogenic and systemic features of fatty liver syndromes, NAFLD and MAFLD, in the individual patient. This or similar accurate descriptive classification systems may facilitate the identification of more homogeneous cohorts of patients to better define the natural course of disease and responses to innovative treatment schedules.

## 7. Conclusions

While research on NAFLD continues to be conducted and published, we are witnessing the dawn of a new era. NAFLD, originally based on the exclusion of competing causes of liver disease (i.e., a disease defined by negation) is increasingly recognized as a truly metabolic disease (hence MAFLD, namely a positive diagnosis). This implicitly takes into account the disappearance of Hepatitis C thanks to the Direct Antiviral Agents and, therefore, the globally changing scenario of risk factors for the development of chronic liver disease [99]. However, MAFLD itself retains elements of ambiguity [66] and words of caution against the risks of prematurely abandoning the old NAFLD definition have been given by eminent experts based on uncertainties regarding definition of metabolic health and given our incomplete understanding of the molecular pathogenesis of disease [100]. This suggests that additional studies will have to ascertain whether MAFLD and NAFLD are equivalent, given that preliminary evidence suggests that NAFLD may specifically identify those individuals with more progressive disease [101] and, therefore, could be more equivalent to the notion of NASH rather than to NAFLD. Challenged by the disappointing findings of many NASH trials [10], what we need is a more accurate definition of NAFLD pathobiology in the individual patient. Proposals to better articulate the diagnosis of NAFLD/MAFLD have recently been formulated [44]. The so called LDE system addresses NAFLD features as seen from the Liver (L), the Determinants of Disease (D) and its extra-hepatic manifestations and complications (E). The LDE system is only one example of how we might better describe our patient population and it is assumed that this will help to improve the so far disappointing attempts to cure NASH. However, this prediction cannot be ascertained unless this or similar classification systems are utilized and assessed in the NAFLD/NASH research arena.

## Figures and Tables

**Table 1:** Classification of NAFLD [27,28,29,30,31,32,33,34]**.**

PRIMARY NAFLD		
	Associated with/predisposing to incident Metabolic Syndrome	
SECONDARY NAFLD		
	Nutritional Disorders	
		total parenteral nutrition, acute starvation
	Abdominal Surgery	
		extensive small bowel resection bilio-pancreatic diversionjejunal bypass
	Drug-Induced	
		diltiazem, aspirin, methotrexate, highly active antiretroviral therapy (stavudine and zidovudine)
	Occupational exposure to organic solvents	
	(Rare) Metabolic Disorders	Hypobetalipoproteinemia, Lipodystrophy, Weber-Christian syndrome, Acute fatty liver of pregnancy, Reyes syndrome and Mauriac syndrome
	VAFLD	
		HIV, HCV
	Common Endocrine Disorders	
		Hypothyroidism, hypogonadism in either sex, GH deficiency, PCOS

## Data Availability

Not applicable.
